# Effects of Gold Nanoparticles Phytoreduced with Rutin in an Early Rat Model of Diabetic Retinopathy and Cataracts

**DOI:** 10.3390/metabo13080955

**Published:** 2023-08-18

**Authors:** Mădălina Moldovan, Ana-Maria Păpurică, Mara Muntean, Raluca Maria Bungărdean, Dan Gheban, Bianca Moldovan, Gabriel Katona, Luminița David, Gabriela Adriana Filip

**Affiliations:** 1 Department of Physiology, Iuliu Hatieganu University of Medicine and Pharmacy, Clinicilor Street, No. 1, 400006 Cluj-Napoca, Romania; papurica.ana.maria@elearn.umfcluj.ro (A.-M.P.); gabriela.filip@umfcluj.ro (G.A.F.); 2 Department of Cell and Molecular Biology, Iuliu Hatieganu University of Medicine and Pharmacy, Pasteur Street, No. 6, 400349 Cluj-Napoca, Romania; muntean.mara@elearn.umfcluj.ro; 3 Department of Pathology, Iuliu Hatieganu University of Medicine and Pharmacy, Clinicilor Street, No. 3-5, 400340 Cluj-Napoca, Romania; maria.bungardean@elearn.umfcluj.ro (R.M.B.); dgheban@gmail.com (D.G.); 4 Department of Pathology, Emergency Clinical Hospital for Children, Motilor Street, No. 41T-42T, 400370 Cluj-Napoca, Romania; 5 Faculty of Chemistry and Chemical Engineering, Babes-Bolyai University, Arany Janos Street, No. 11, 400028 Cluj-Napoca, Romania; bianca.moldovan@ubbcluj.ro (B.M.); gabik@chem.ubbcluj.ro (G.K.); luminita.david@ubbcluj.ro (L.D.)

**Keywords:** diabetic retinopathy, cataracts, gold nanoparticles, Rutin, antioxidant, early, incipient

## Abstract

Diabetic retinopathy (DR) and cataracts (CA) have an early onset in diabetes mellitus (DM) due to the redox imbalance and inflammation triggered by hyperglycaemia. Plant-based therapies are characterised by low tissue bioavailability. The study aimed to investigate the effect of gold nanoparticles phytoreduced with Rutin (AuNPsR), as a possible solution. Insulin, Rutin, and AuNPsR were administered to an early, six-week rat model of DR and CA. Oxidative stress (MDA, CAT, SOD) was assessed in serum and eye homogenates, and inflammatory cytokines (IL-1 beta, IL-6, TNF alpha) were quantified in ocular tissues. Eye fundus of retinal arterioles, transmission electron microscopy (TEM) of lenses, and histopathology of retinas were also performed. DM was linked to constricted retinal arterioles, reduced endogen antioxidants, and eye inflammation. Histologically, retinal wall thickness decreased. TEM showed increased lens opacity and fibre disorganisation. Rutin improved retinal arteriolar diameter, while reducing oxidative stress and inflammation. Retinas were moderately oedematous. Lens structure was preserved on TEM. Insulin restored retinal arteriolar diameter, while increasing MDA, and amplifying TEM lens opacity. The best outcomes were obtained for AuNPsR, as it improved fundus appearance of retinal arterioles, decreased MDA and increased antioxidant capacity. Retinal edema and disorganisation in lens fibres were still present.

## 1. Introduction

Research on ocular complications of diabetes mellitus (DM), including diabetic retinopathy (DR) and cataracts (CA), is currently shifting its focus towards early detection and therapy [1,2]. This is a shift from what is now regarded as the standard method, which primarily focuses on late-phase treatment. These strategies have a painful and limited curative ability for DR, and pose a significant economic burden for CA [3,4]. Moreover, CA surgery, despite being a customary practice, was found to be associated with an increased risk of DR development in diabetic patients [5]. The transition from advanced-stage management to early-stage prevention is being made possible, firstly by advancements in screening [6,7]. 

Numerous studies have emphasized the pressing issue of timely detection at subclinical stages, defined by the presence of morphophysiological alterations with limited characteristic symptoms. One study on diabetic patients [2] described peripheral spoke-like lesions on the eye lens through slit-lamp examination, typically associated with cortical CA (a type often linked to diabetes). These lesions only became symptomatic with accumulation in the centre of the lens, leading to impaired vision, correctable by surgery. Likewise, subclinical abnormalities in DR resulting from early microvascular and neuronal degeneration were measured using spectral domain optical coherence tomography and optical coherence tomography angiography [8].

Thus, the impact of subclinical diagnosis by various screening methods could be increased by subsequent treatment. At present, lifestyle changes are primarily recommended, specifically blood glucose management. A metanalysis study [9] found that, despite being guideline advocated, strict glycaemic control could not be correlated to significant benefits against microvascular complications. To address this research gap, recent studies have postulated the use of plant-derived antioxidants as effective remedies for diabetes-induced complications. This can be justified by the existing understanding of pathophysiology. According to Brownlee’s hypothesis [10], there is a singular common mechanism that causes both diabetes and its complications—increased production of reactive oxygen species (ROS). This model states that the typical diabetes-linked reduced amount of insulin and/or its effects trigger a systematic chain of events. Firstly, the ensuing hyperglycaemia stimulates an increased flux through the major pathways responsible for diabetes damage—polyol, hexosamine, protein kinase C (PKC), advanced glycation end-product formation (AGEs) and activation of Poly(ADP-ribose) polymerase. Secondly, vulnerable glial cells, such as astrocytes, Müller cells and microglia, enter a state of oxidative stress, which is coupled with an increase in the secretion of proinflammatory cytokines, TNF-α, IL-1β, and IL-6 [11]. Furthermore, it has been proposed [12] that the retinal glia hosts a resident renin-angiotensin system (RAS), which produces local Angiotensin II (Ang II). Intraocular levels of Ang II have been shown to rise in diabetes and were associated with an increased inflammatory and oxidative stress response. Ang II has also been found to stimulate the retinal microglia through the Angiotensin type 1 receptor (AT1), leading to a decrease in retinal blood flow, independent of systemic changes [13]. Similarly, RAS modulators have demonstrated anticataract properties, possibly by inhibiting the AT1-mediated production of ROS [14].

A variety of antioxidant plant compounds have been suggested as potential treatments for diabetic ocular complications, each presenting individual strengths and weaknesses. Among these compounds, Rutin, a flavonol from the flavonoid subclass of dietary polyphenols, shows promising properties for managing DM. Rutin is known for its antioxidant and anti-inflammatory abilities [15]. Additionally, a recent study has demonstrated that Rutin can act as an inhibitor of AT1 receptors [16]. Moreover, it can lower glycaemia by inhibiting carbohydrate absorption and gluconeogenesis, and it can promote insulin secretion and the cellular uptake of glucose [15]. This compound has also been shown to decrease serum triglycerides, LDL, and VLDL, with high HDL levels in experimental models [15].

The main disadvantage of Rutin is its low bioavailability, attributed to its highly hydrophilic nature, which hinders diffusion through cell membranes. After ingestion, Rutin is hydroxylated to quercetin, a compound quickly metabolised in the body, resulting in limited bioavailability [17]. 

Delivering therapeutic agents to the eye poses an additional challenge. As such, barriers exist for both topical administrations, the tear layer and corneal epithelium with tight junctions [18], and systemic delivery, including the blood–aqueous barrier and blood–retina barrier [19]. 

Considering the systemic nature of diabetes, and Rutin’s multiorgan action, oral administration, together with a nanotechnology-based formulation, were proposed to overcome delivery and bioavailability issues. Nanoparticles are accepted as efficient drug delivery systems for both lens and retinal pathologies. Gold nanoparticles (AuNPs) have been used in previous medical studies due to their ease of functionalisation with various active molecules [20]. A recent study [21] demonstrated significant uptake of AuNPs functionalised with resveratrol, a stilbene from the subclass of dietary polyphenols, in ocular lens epithelial cells, in both in vitro and in vivo models of CA, with no dose-dependent toxicity. Similarly, improvements were demonstrated in a Wistar rat model of diabetic retinopathy when treated with AuNPs phytoreduced with resveratrol [22]. Other authors [23] revealed increased in vitro glucose uptake by adipocytes using AuNPs prepared with vicenin-2, a compound from the same flavonoid subclass as Rutin. 

In the present study, we hypothesized that the administration of gold nanoparticles conjugated with Rutin (AuNPsR) in an early rat model of DR and CA may present beneficial therapeutic effects. The effects were evaluated by oxidative stress investigation in serum and ocular tissues, and inflammatory cytokines levels in eye homogenates. Eye fundus assessment of retinal arterioles, transmission electron microscopy (TEM) of eye lenses, and histopathological examination of retinas were also performed. 

## 2. Materials and Methods

### 2.1. Reagents

Tetrachloroauric acid trihydrate, 2-thiobarbituric acid, Bradford reagent, sodium hydroxide, and Folin–Ciocalteu reagent were obtained from Merck (Darmstadt, Germany). O-phthalaldehyde, osmium tetroxide and glutaraldehyde were purchased from Sigma–Aldrich (Taufkirchen, Germany). IL1β, TNFα, and IL6 were measured in eye homogenates by ELISA assays using the Elabscience ELISA kits (Houston, TX, USA), according to the producer instructions. Results were expressed as pg/mg protein. 

### 2.2. Gold Nanoparticles Synthesis and Characterisation

The synthesis of AuNPsR was carried out as follows: to a mixture of 61 mg Rutin and 100 mL distilled water, 2 M aqueous solution of NaOH was added dropwise (approximately 4 mL), until Rutin was totally dissolved, and the colour of the obtained solution turned yellow-orange. An amount of 100 mL of 1 mM tetra chloroauric solution was added over the Rutin solution and the mixture was stirred at room temperature for one hour. The obtained AuNPsR were purified via centrifugation at 10,000 rpm followed by washing of the resulting pellet twice with distilled water. The pellet was resuspended and used for biological determination. The obtained AuNPsR were characterised using classical methods. UV-Vis spectroscopy was applied to follow the progress of the reaction; to this end, a Perkin Elmer Lambda 25 spectrometer was used. The absorbance of gold colloidal solution was scanned between 300 and 800 nm, in a 1 cm quartz cuvette, with distilled water being used as blank. Transmission electron microscopy (TEM), by using a Hitachi H-7650 transmission microscope, made possible the morphological characterisation of AuNPsR. The ImageJ 1.53 t [24] software was used to measure the mean size of the synthesized AuNPsR, from at least 100 AuNPsR. The zeta potential and hydrodynamic diameter of AuNPsR were assessed using a Malvern Zetasizer Nanoseries compact scattering spectrometer. The crystal structure and the crystalline grain size of the obtained gold nanoparticles were analysed via X-ray crystallography. X-ray diffraction (XRD) data were acquired with a Smart Lab Rigaku diffractometer with a graphite monochromator with Cu-Ka radiation (k ¼ 1:54 Å) X-ray source: Anode Cu, 9 kW at room temperature over the 2Theta range from 10 to 90 degrees, with a 0.01-degree step. For the Rietveld refinement, the Integrated X-ray Powder Diffraction (PDXL) software was used.

### 2.3. Experimental Design

The study adhered to the ethical standards regarding animal research and received approval from the University Ethical Board and the Veterinary and Food Safety Direction (project authorisation no. 294/09.03.2022). Sixty-eight Wistar albino female rats, three months old, weighing 300 ± 10 g, were provided by the Experimental Animal Facility of Iuliu Hatieganu University of Medicine and Pharmacy in Cluj-Napoca, Romania. Rats were housed in cages under standard environmental conditions, with a temperature of 21 ± 2 °C, a relative humidity of 55% ± 5%, and a 12 h light/12 h dark cycle. Conventional food and water were provided ad libitum.

**Figure 1:**
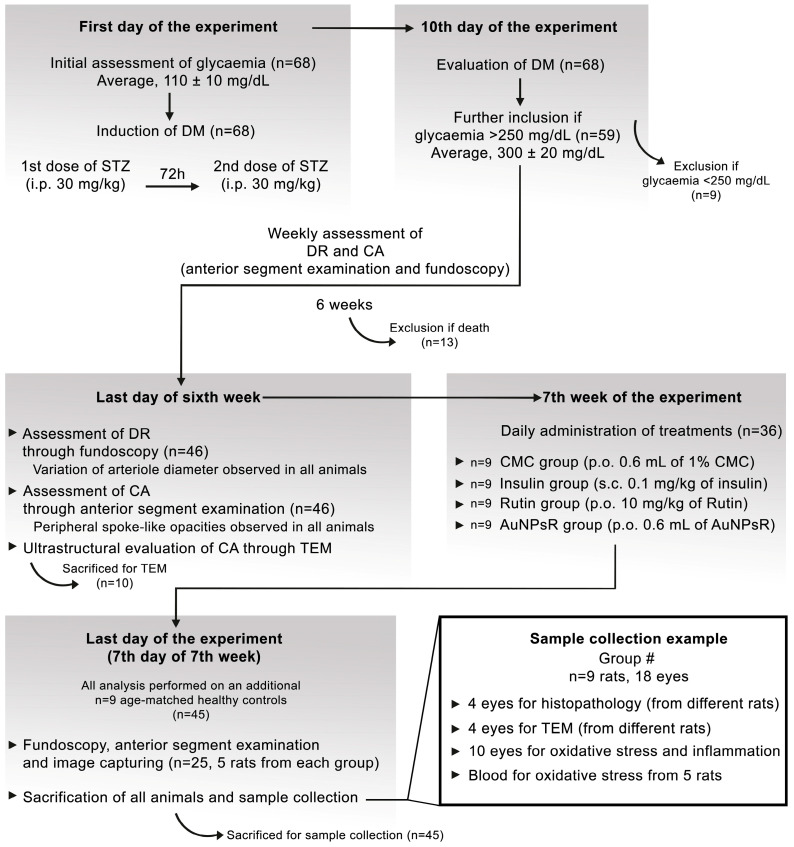
Illustrative representation of the experimental design; DM = diabetes mellitus; STZ = streptozotocin; i.p. = intraperitoneal; DR = diabetic retinopathy; CA = cataracts; TEM = transmission electron microscopy; CMC = carboxymethylcellulose; s.c. = subcutaneous; p.o. = per os.

The experiment consisted of three stages: induction of diabetes, development of incipient ocular complications, and administration of treatment. The experimental design is illustrated in Figure 1.

Prior to the induction of DM, blood samples were drawn from 68 animals to assess glycaemic levels, which were found to be within the normal range (average of 110 ± 10 mg/dL). To induce DM, two doses of streptozotocin (STZ) were administered to all 68 rats by intraperitoneal injection: 30 mg/kg STZ on day zero and 30 mg/kg STZ after 72 h. Animals were included in the study if their blood glucose levels exceeded 250 mg/dL on the seventh days after the last dose of STZ. At this point, nine rats were excluded because their glycaemic level was below 250 mg/dL. The average glycaemic value obtained for the remaining 59 animals was 300 ± 20 mg/dL.

**Figure 2:**
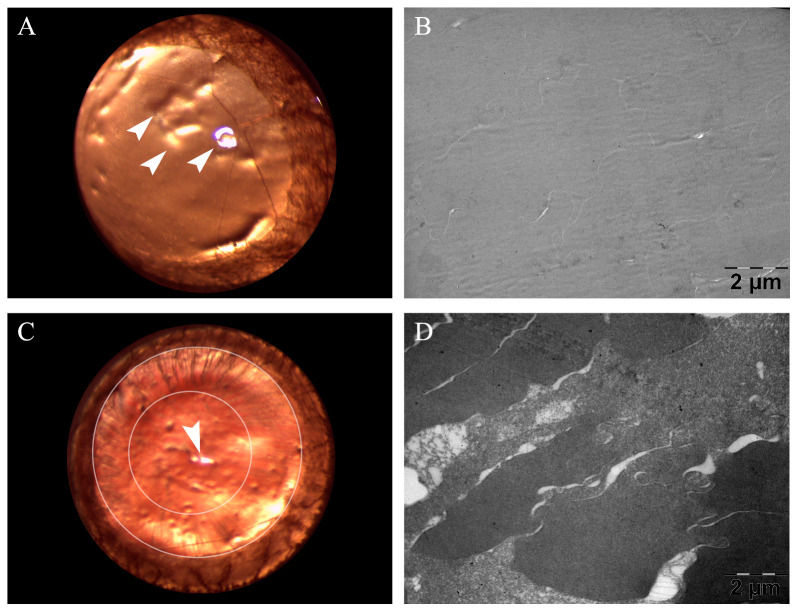
Side-by-side images obtained from a healthy rat and a six-week diabetic rat; (**A**) anterior segment photography of healthy eye lens. (**B**) Transmission electron microscopy (TEM) micrograph of healthy eye lens with lens fibres tightly packed together, separated by thin spaces. (**C**) Anterior segment photography depicting peripheral spoke-like opacities of incipient cataracts; the larger oval outlines the internal limit of the iris, while the smaller oval outlines the internal border of cataracts lesions, more visible in the upper left quadrant (from eleven to one clockwise). (**D**) TEM micrograph of eye lens with notable lens fibre disorganisation, characteristic of incipient cataracts. Arrowheads point towards microscope reflection, not to be confused with central opacity.

In the following six weeks after induction of DM, weekly anterior segment examination and fundoscopy were performed to assess the incipient development of CA and DR. In this timeframe, 13 animals were excluded due to death. On the last day of the sixth week, 46 rats survived and were further analysed. Early DR was defined through variations in retinal arteriole diameter and was detected in all surviving animals. Similarly, all animals exhibited the first signs of CA formation, including swollen fibres and subcapsular opacities, according to a previously described grading system of lens opacity [25]. Ten randomly selected diabetic animals were sacrificed and used for the ultrastructural evaluation of CA through TEM. Additionally, the same evaluation was performed for age-matched healthy rats, which were considered controls. A side-by-side view of the anterior segment photography and the corresponding TEM micrographs is presented in Figure 2. 

Subsequently, 36 animals with signs of incipient diabetic ocular complications were included in the study and were randomly divided in the following four treatment groups (nine animals/group): CMC group (0.6 mL/day of 1% carboxymethylcellulose vehicle solution), Insulin group (0.1 mg/kg of insulin), Rutin group (10 mg/kg/day), and AuNPsR group (0.6 mL/day of AuNPsR). A fifth group with nine healthy age-matched animals, without DM and treated only with CMC, was considered the Control group. Treatments were administered daily for seven days, via oral gavage, between 7 a.m. and 8 a.m., except for insulin, which was administered subcutaneously.

After seven days of treatment, five randomly selected animals/group were completely sedated in order to perform fundoscopy, anterior segment evaluation, and image collection. Then, all specimens (n = 45) were sacrificed by overdose as to not damage the eye from the increased pressure. For microscopic studies, a total of eight eyes, from four animals/group, were enucleated. These eight eyes/group were distributed evenly between histopathology and TEM processing. Specifically, each of the four animals contributed one eye to TEM processing and another eye to histopathology processing. This approach ensured that all eyes subjected to microscopic studies were sourced from distinct animals, randomly chosen from each group. From five animals/group, blood was used to assess oxidative stress levels, whilst their ten eyes were collected for biochemical analysis. 

All eyes were collected using a technique specifically developed to maintain structural integrity. The soft tissues overlaying the skull were removed. Then, an incision was made along the frontonasal suture, and the dorsal part of the skull was gently lifted. This exposed the ocular globes and their respective optic nerves, which were easily enucleated and further processed.

### 2.4. Fundoscopy Examination

Fundus photographs were captured using a Leica Microsystems M320 T Surgical Training Microscope. Pupils were dilated with a drop of 1% tropicamide, and the vibrissae were trimmed to prevent them from obstructing the photographs. During the procedure, eyelids were completely retracted, and a mound of propylene glycol water-based viscous gel was applied to the external portion of the rodent’s eye. A glass microscope slide was placed on top of the gel, and gentle pressure was exerted to flatten the cornea. The gel prevents the image from distorting and provides a practical alternative to liquid oil in this situation. The position of the specimen and the pressure applied to the glass slide were permanently adjusted to achieve the proper focus. The specimen’s position and angle were altered accordingly to examine different areas of the fundus. All photographs were captured using the built-in camera of the surgical microscope.

### 2.5. Fundus Photography Processing and Analysis

Fundus photographs were analysed using ImageJ version 1.53 k [24]. Firstly, arterioles were differentiated from venules based on the anatomical model described by McLenachan et al. [26]. Thus, an alternating pattern was observed, with each arteriole being situated next to a venule. Following the approach outlined by Miri et al. [27], the darker red vessels were considered as venous in nature. 

To facilitate the visualisation of red colour variance, all photographs were imported into ImageJ, converted to the RGB format, and then split into greyscale colour channels. For the red channel, brighter greys indicated higher red amounts, while darker greys meant lower red amounts or no red. As a result, arterioles, associated with a bright red colour due to oxygenated blood, appeared as washed-out brighter grey, while venules, associated with a dark red colour due to deoxygenated blood, appeared as clearly distinguishable dark grey. This facilitated successful arteriole isolation.

**Figure 3:**
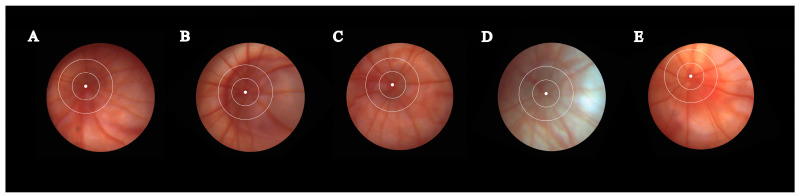
A representative fundus photography from each group, in controls (**A**) and in rats with six weeks of diabetes and one week of treatment as follows: CMC (carboxymethylcellulose) (**B**), insulin (**C**), Rutin (**D**), AuNPsR (gold nanoparticles phytoreduced with Rutin) (**E**). The selected region for measuring retinal arteriole diameter is defined as the area enclosed between the smaller circle and the larger circle.

For measurements, the raw fundus photographs were used, as depicted in Figure 3. Fundus photographs were obtained from both eyes of five randomly selected animals of every group. For each eye, three arterioles were measured, resulting in 30 measurements for every group (three arterioles × two eyes per animal × five animals per group). The selected measurement area was within a disc diameter of 0.5 to 0.25 from the edge of the fundoscopy disc margin. This area was chosen to account for the curvature of the eye and to ensure measurement consistency across photographs. A central line was drawn following the long axis of the vessel. To assess diameter, five distinct measurement lines perpendicular to the central line were created. The width of each measurement line was calculated, and the five values were averaged. 

### 2.6. Oxidative Stress Investigation and Inflammation Assessment

Oxidative stress was evaluated in serum and eye homogenates by quantification of malondialdehyde (MDA), as a marker of lipid peroxidation, using Conti’s method [28]. Additionally, catalase (CAT) activity was measured through Pippenger’s method [29]. Superoxide dismutase (SOD) activity was assessed through the method described by Beauchamp and Fridovich [30]. Inflammation from eye homogenates was evaluated via ELISA tests, and the results were expressed as pg/mg protein.

### 2.7. Retina Histopathological Examination

Extraocular tissues were excised immediately after enucleation, to ensure proper penetration of the fixative. Then, the eyes underwent a two-step fixation process. For this purpose, a 10% formaldehyde solution was utilized, at ten times the volume of the tissue being studied. For the first 24 h, the ocular globes were submerged in the fixation solution, as a whole. Subsequently, they were briefly removed from the fixative, and sectioned in half, along the anatomical sagittal plane (with the blade placed perpendicular to the superior and inferior rectus muscles). Then, the obtained halves were fixed for a second 24 h period, in the same solution. Through employing a preliminary fixation of the entire globe, collapse upon halving was avoided. Ultimately, samples were embedded in paraffin and sectioned at 5 μ, then stained with haematoxylin-eosin and examined using an Olympus BX43F light microscope (Olympus, Tokyo, Japan). Pictures of representative areas were captured using the microscope mounted camera (Olympus UC30 camera with the Olympus U-CMAD3 adapter).

### 2.8. Eye Lens Transmission Electron Microscopy

Following enucleation, eye lenses were removed as swiftly as possible. The ocular globe was held in place with toothed microsurgical forceps whilst the cornea was removed along the limbus, by gliding two surgical blades against each other and in opposing directions. After extraction, eye lenses were first fixed in a 2.7% glutaraldehyde solution in 0.1 M phosphate buffer, and then they were cut in halves. Afterward, samples were washed four times, in the same buffer. Subsequent to postfixation with 1.5% osmium tetroxide (OsO4) in 0.15 M phosphate buffer, eye lenses underwent dehydration in a series of acetone solutions of increasing concentrations (from 30%, up to 100%), infiltration and embedding in EMbed 812. Using a Diatome A382 diamond knife (Diatome, Hatfield, USA), 70–80 nm thick ultrathin sections were obtained on a Bromma 8800 ULTRATOME III (LKB, Stockholm, Sweden). These sections were then collected on 300 mesh copper grids, contrasted with uranyl acetate and lead citrate, and examined at 80 kV using a JEOL JEM-100CX II transmission electron microscope (JEOL, Tokyo, Japan). Images were captured with a MegaView G3 camera, equipped with a Radius 2.1 software (both from Emsis, Münster, Germany).

### 2.9. Eye Lens TEM Photography Processing and Analysis

To assess opacity modifications in eye lenses using TEM micrographs, an adapted protocol based on Wirahadikesuma et al. was implemented [31]. Micrographs were analysed using ImageJ version 1.53 k [24].

Twenty micrographs from each group were examined. For each micrograph, 20 regions of interest were measured by two blinded researchers, resulting in a total of 800 measurements for each group (20 photos per group × 20 measurements per micrograph × two independent researchers). Regions of interest were selected based on the homogeneity of lens tissue and were defined as a square area, with the calculated side length of 365 pixels, which would provide 95% coverage of each micrograph. As the measurement unit was not significant to our outcome, the standard pixel unit was utilised.

To measure pixel density, the .tif micrographs were converted to the commonly used 8-bit integer format, yielding a possible range of pixel densities from zero to 255. Following Ansel Adam’s Zone System, zero represents pure black, and 255 represents pure white. Considering that the images are micrographs, where pure black corresponds to complete electron density, such as a perfect saturation of the employed TEM fixative and contrasting agent (OsO4), zero was considered the maximum opacity achievable by our tissue. Measurements from the control group were used as a reference. Hence, the maximum pixel density obtained in the control group was considered the transparency standard, with a value of 166.949 pixels/area. To facilitate visualisation of opacity measurements, the following formula was applied to convert pixel density to a percentage of opacity,

(255 − x)/255 × 100.(1)

**Figure 4:**
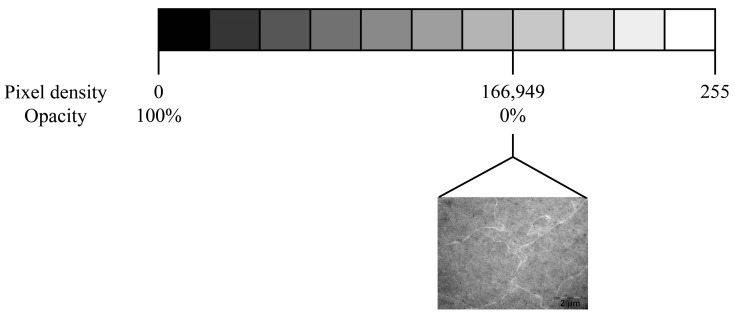
Depiction of Ansel Adam’s Zone System according to a greyscale gradient, which ranges from zero pixels, or the equivalent of pure black, to 255 pixels, or the equivalent of pure white. For our experimental purpose of evaluating eye lens opacity using transmission electron microscopy micrographs, 100% opacity was attributed to zero pixels, and 0% opacity, or standard transparency, to 166.949 pixels. This value corresponds to the presented micrograph of a subject from the control group, which demonstrated the highest transparency.

Consequently, 100% opacity would correspond to zero pixels/area (maximum opacity), and 0% opacity would correspond to 166.949 pixels/area (minimum opacity or maximum transparency achievable). This conversion is depicted in Figure 4. 

Any heterogeneity caused by CA-induced morphological modifications would be indicated by higher electron density and consequently a tendency towards increased opacity.

### 2.10. Statistical Analysis

Data were analysed using GraphPad Prism version 9.0.0 for Windows, GraphPad Software, San Diego, CA USA, www.graphpad.com, accessed on 15 May 2021. All multi-group assessments were performed using the Kruskal–Wallis test for not normally distributed data. Outliers of TEM micrographs were identified using Robust regression and Outlier removal (ROUT) with Q set at the default 1%. To describe the quantitative data from arteriole diameter and eye lens opacity investigations, the minimum and maximum values, median, and interquartile range (Q1–Q3, the range between the 25th percentile and the 75th percentile) were graphed. Additionally, quantitative data from serum oxidative stress, eye tissue oxidative stress and inflammation examinations, was described through mean and standard deviation. A *p*-value equal to or lower than 0.05 was considered statistically significant. Photos were analysed using ImageJ 1.53 k [24].

## 3. Results

### 3.1. Characterisation of Gold Nanoparticles Functionalised with Rutin

**Figure 5:**
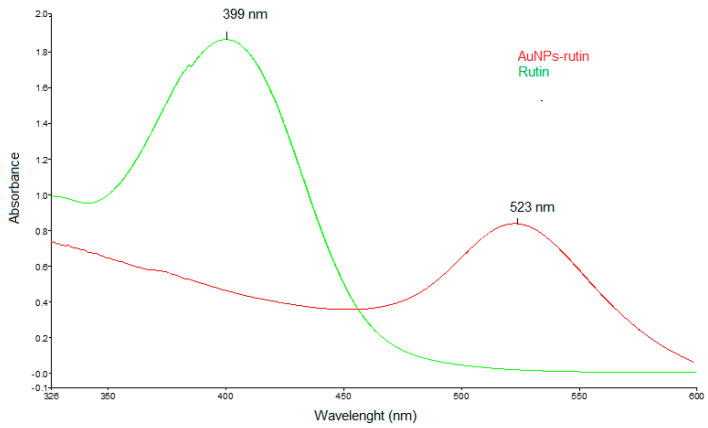
UV-Vis spectra of Rutin and gold nanoparticles phytoreduced with Rutin (AuNPsR).

In order to avoid the use of toxic solvents and the production of harmful waste, an environmentally friendly synthesis method was applied for the production of AuNPsR. Rutin was successfully used to reduce the gold ions at room temperature and also to prevent the aggregation of the synthesized nanoparticles by acting as a capping agent at their surface. The UV-Vis spectrophotometry was used to monitor the synthesis of AuNPsR and to confirm their obtaining after reduction of the gold ions from the HAuCl_4_ solution, obtaining that was first visually confirmed by the colour change from yellow to purple-red. Figure 5 presents the recorded UV-Vis spectra of the Rutin solution and AuNPsR colloidal solution. It is easy to observe that the Vis typical absorption band of Rutin at 399 nm [32] disappeared during the progress of the synthesis, and the AuNPsR characteristic surface plasmon resonance SPR band at λ = 523 nm appeared [33].

**Figure 6:**
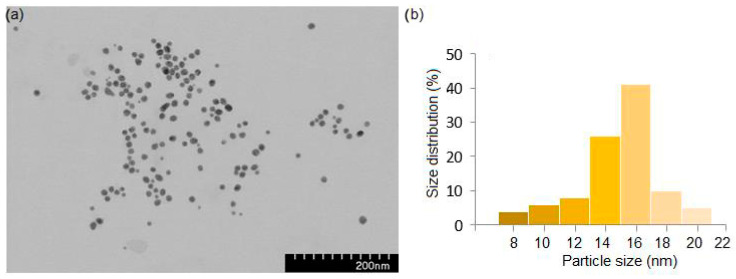
TEM image (**a**) and size distribution (**b**) of gold nanoparticles phytoreduced with Rutin (AuNPsR).

The morphology, diameter, and size distribution of the AuNPsR were analysed using transmission electron microscopy (TEM). The TEM image, shown in Figure 6a, revealed that the obtained AuNPsR were mostly spherical in shape, presenting homogenous size distribution and an average diameter (Figure 6b) of 15 nm. 

**Figure 7:**
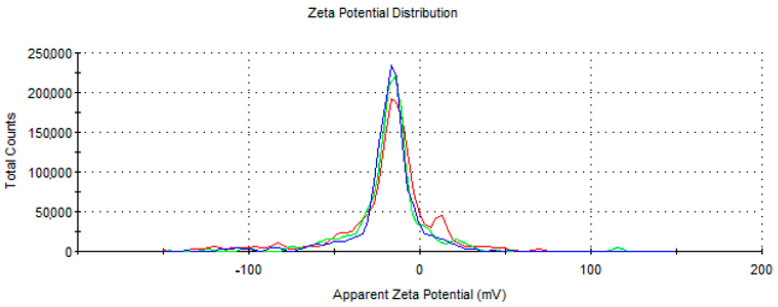
Zeta potential of gold nanoparticles phytoreduced with Rutin (AuNPsR). There are three distinct measurement sets, each consisting of thirty individual runs. Every coloured line corresponds to a singular measurement set.

Dynamic light scattering (DLS) experiments allowed us to evaluate the stability of the AuNPsR solution and their surface charge and also to analyse their hydrodynamic diameter. The obtained negative zeta potential value, measured to be −19.0 eV (Figure 7), suggests that the resulting colloidal solution of AuNPsR presents a rather good stability. The negative surface charge of the AuNPsR is due to the Rutin molecules negatively charged that are present at the surface of the nanoparticles, which confer them a good stability [34].

The hydrodynamic diameter of the AuNPsR was found to be 78.48 nm.

**Figure 8:**
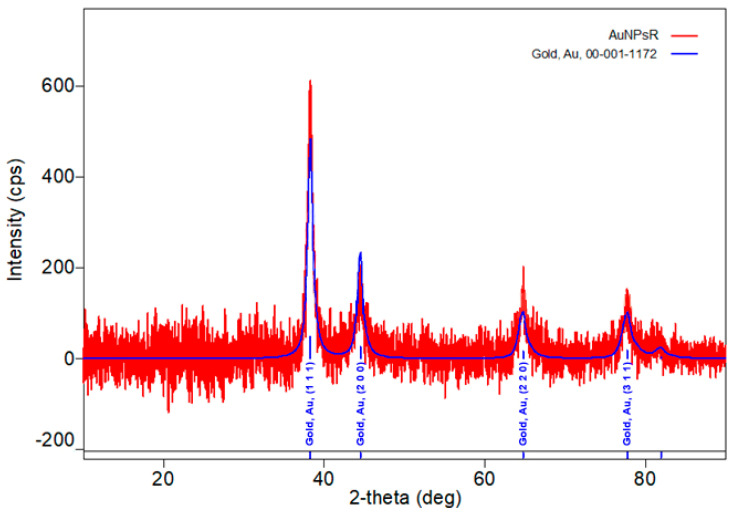
X-ray diffraction pattern of synthesized gold nanoparticles phytoreduced with Rutin (AuNPsR).

X-ray diffraction analysis was used to determine the crystal structure of the obtained gold nanoparticles. Figure 8 presents the XRD diffractogram of AuNPsR.

The presence of the four characteristic peaks at 2θ values of 38.28°, 44.52°, 64.74°, and 77.90°, corresponding to the reflection planes of (111), (200), (220), and (311) of faced centred cubic gold was observed (identification ICDD DB card 00-001-1172). The Williamson–Hall method was used to measure the crystallite size, which was found to be 58 Å, thus confirming the formation of gold nanoparticles.

### 3.2. Fundoscopy Examination

**Figure 9:**
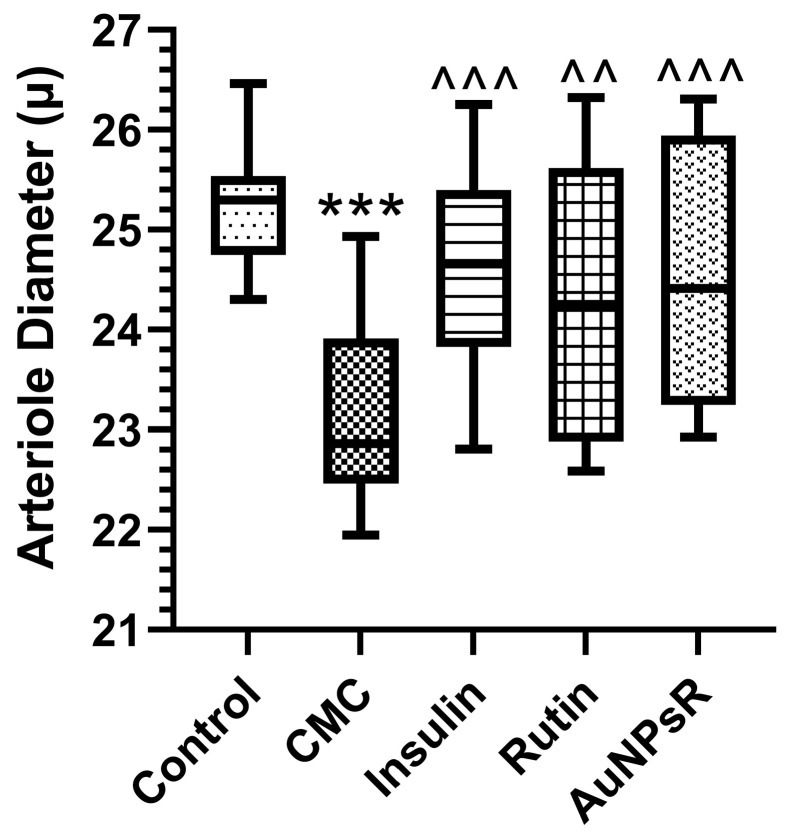
Retinal arterioles variation of diameter in Control group, and in rats with six-week diabetes, followed by one week of treatments: carboxymethylcellulose (CMC), insulin, Rutin, and gold nanoparticles phytoreduced with Rutin (AuNPsR). Parameters are expressed as minimum and maximum values, median, and interquartile range (Q1–Q3, the range between the 25th percentile and the 75th percentile), with *** *p* < 0.001 compared to Control group; ^^ *p* < 0.01, ^^^ *p* < 0.001 compared to CMC group.

To evaluate arteriole diameter differences between groups, the Kruskal–Wallis test for not normally distributed data was used. The results are depicted in Figure 9.

In terms of arteriole diameter, diabetic specimens from CMC group demonstrated a significant vasoconstriction compared to controls (*p* < 0.001). The administration of insulin to diabetic rodents resulted in a statistically significant increase in arteriole diameter compared to CMC (*p* < 0.001). The same pattern was obtained after treatment of diabetic subjects with Rutin (*p* < 0.01). The administration of AuNPsR enhanced the diameter of arterioles for diabetics compared to CMC (*p* < 0.001). All treatments used proved a good effect on the retinal arteriole diameter. It is worth highlighting that administration of Rutin and AuNPsR displayed a significant spread of values in relation to their respective median lines.

### 3.3. Blood and Eye Tissue Oxidative Stress Investigation 

**Figure 10:**
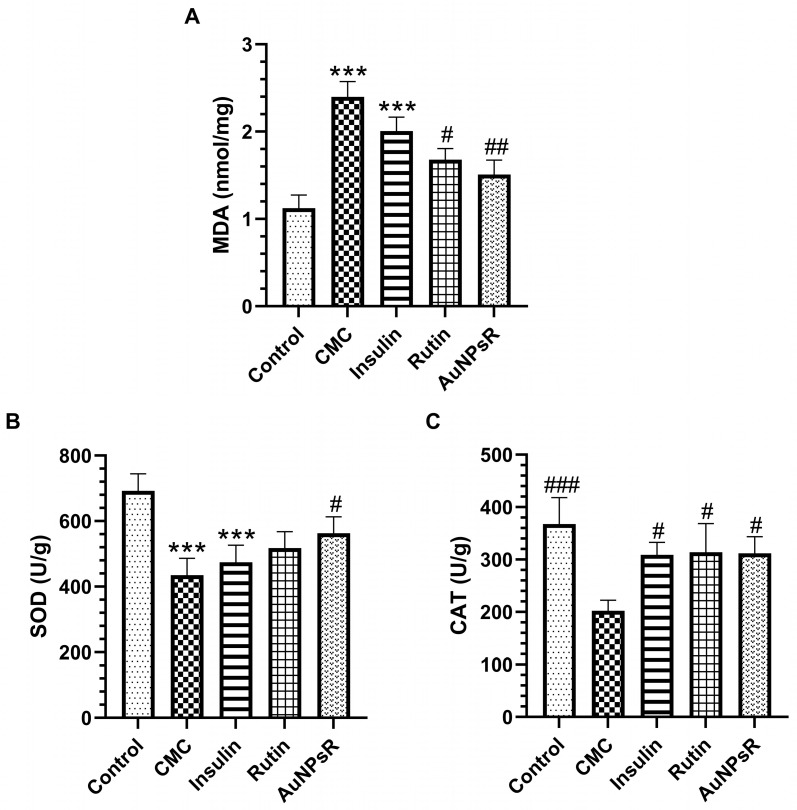
Blood oxidative stress assessment. (**A**) Malondialdehyde (MDA) levels, (**B**) superoxide dismutase (SOD) and (**C**) catalase (CAT) activities in controls, and in diabetic animals treated with CMC (carboxymethylcellulose), insulin, Rutin, and AuNPsR (gold nanoparticles phytoreduced with Rutin). Parameters are expressed as mean and standard deviation, with *** *p* < 0.001 compared to Control group; # *p* < 0.05, ## *p* < 0.01, and ### *p* < 0.001 compared to CMC group.

Results from the oxidative stress investigation of blood samples are presented in Figure 10. Blood sample analysis revealed that animals with DM and treated with CMC had high levels of MDA compared to controls (*p* < 0.001). Similar results were obtained in the insulin treatment group. The administration of Rutin decreased blood MDA levels compared to CMC group (*p* < 0.05). Accordingly, the administration of AuNPsR in diabetic animals lowered MDA level in serum compared to CMC (*p* < 0.01). Notably, AuNPsR administration did not show statistical difference when compared to Rutin (*p* > 0.05). The activity of SOD in the CMC group decreased compared to controls (*p* < 0.001). Additionally, similar results were observed for the insulin-treated group. Only AuNPsR administration increased SOD activity compared to CMC group (*p* < 0.05). Catalase (CAT) activity diminished in CMC-treated rats when compared to controls, *p* < 0.001. Moreover, all treatments improved CAT activity when compared to the CMC group, as follows: insulin, *p* < 0.05; Rutin, *p* < 0.05; AuNPsR, *p* < 0.05.

**Figure 11:**
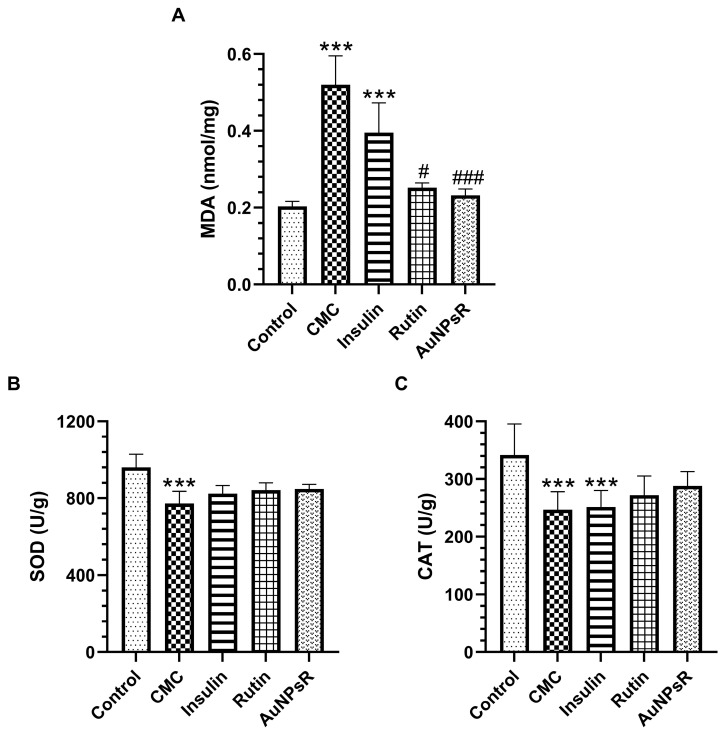
Oxidative stress parameters in eye tissues homogenates. (**A**) Malondialdehyde (MDA) levels, (**B**) superoxide dismutase (SOD), and (**C**) catalase (CAT) activities, in controls and in rats with DM (diabetes mellitus) and treated with CMC (carboxymethylcellulose), insulin, Rutin, and AuNPsR (gold nanoparticles phytoreduced with Rutin). The parameters are expressed as mean and standard deviation, with *** *p* < 0.001 compared to Control group; # *p* < 0.05, and ### *p* < 0.001 compared to CMC group.

The oxidative stress investigation outcomes of eye tissue homogenate are depicted in Figure 11. Malondialdehyde (MDA) levels in the eye homogenate increased significantly in the CMC group compared to controls (*p* < 0.001). Notably, similar results were observed in the insulin treatment group. Rutin administration decreased ocular MDA levels compared to CMC (*p* < 0.05). Similarly, AuNPsR-treated diabetics showed an important decrease in the ocular MDA level when compared to the CMC group (*p* < 0.001). Ocular superoxide dismutase (SOD) activity diminished in the CMC-treated diabetes group, when compared to controls (*p* < 0.001). The compounds administered had no favourable effects regarding ocular SOD activity. Eye homogenate catalase (CAT) activity decreased statistically in both CMC (*p* < 0.001) and insulin (*p* < 0.001) groups, when compared to controls.

### 3.4. Eye Tissue Inflammation Investigation 

**Figure 12:**
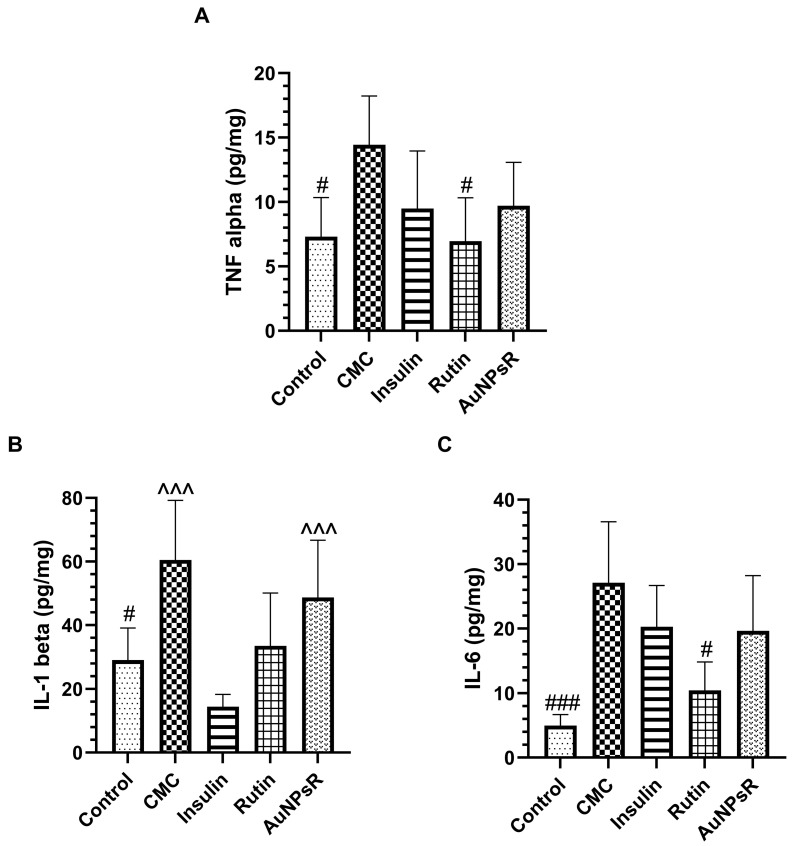
Proinflammatory cytokines levels in eye homogenates, (**A**) TNF alpha, (**B**) IL-1 beta, and (**C**) IL-6, in controls and in rats with DM and treated with CMC (carboxymethylcellulose), insulin, Rutin, and AuNPsR (gold nanoparticles phytoreduced with Rutin). Parameters are expressed as mean and standard deviation, with # *p* < 0.05, ### *p* < 0.001 compared to CMC group; ^^^ *p* < 0.001 compared to Insulin group.

Proinflammatory cytokines levels measured from eye homogenate are graphed in Figure 12. In the CMC group, TNF alpha secretion increased significantly when compared to controls (*p* < 0.05), while Rutin administration reduced TNF alpha levels, when compared to the CMC group (*p* < 0.05). Accordingly, IL-1 beta levels were significantly enhanced in CMC-treated diabetic animals, when compared to controls (*p* < 0.05), while insulin administration showed a notable decrease in IL-1 beta (*p* < 0.001). Remarkably, AuNPsR administration demonstrated a marked increase in IL-1 beta levels, when compared to insulin-treated diabetic animals (*p* < 0.001). Moreover, out of all treatments administered, AuNPsR showed the highest levels of IL-1 beta. In a similar manner to previous inflammatory cytokines, CMC-treated diabetics showed a statistical increase in IL-6 levels, when compared to controls (*p* < 0.001). Both insulin and AuNPsR yielded similar results, with a decrease in IL-6, compared to the CMC group, but without statistical significance. A favourable outcome was noted only in the Rutin group, which showed a statistically significant decrease in IL-6 when compared to CMC administered diabetics (*p* < 0.05).

### 3.5. Retina Histopathological Investigation

**Figure 13:**
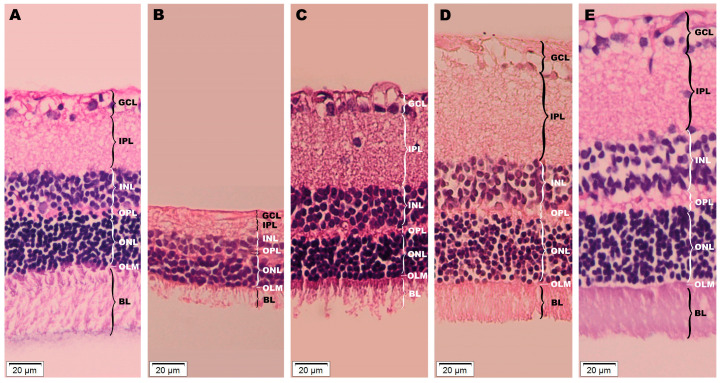
Histopathological investigation of retinas from (**A**) healthy specimens, and from six-week diabetic animals, with a subsequent one week of the following treatments: (**B**) CMC (carboxymethylcellulose), (**C**) insulin, (**D**) Rutin, (**E**) AuNPsR (gold nanoparticles phytoreduced with Rutin); a significant difference in overall retinal thickness is visible, with varying width for each individual layer; increasing levels of edema are perceptible, minimal for insulin (**C**), moderate for Rutin (**D**), and advanced for AuNPsR (**E**); layers of retinas from each group are delineated: GCL (ganglion cell layer), IPL (inner plexiform layer), INL (inner nuclear layer), OPL (outer plexiform layer), ONL (outer nuclear layer), OLM (outer limiting membrane), and BL (bacillary layer).

Images from the histopathological investigation of retina samples are illustrated in Figure 13.

In terms of global changes, a difference in average retinal thickness was observed. In comparison to the CMC group (115.2 ± 4.2 μ), all treatments increased this parameter: insulin (125 ± 1.2 μ), Rutin (179.4 ± 2.6 μ), and AuNPsR (204.4 ± 2.4 μ). In terms of neural-related changes, the width of the individual retinal layers was measured. Our target treatments, Rutin and AuNPsR, showed a higher width in all cell body layers, when compared to CMC: ganglion cell layer (CMC, 7.4 ± 0.8 μ; Rutin, 7.9 ± 0.2 μ; AuNPsR, 10.9 ± 0.1 μ), inner nuclear layer (CMC, 18.5 ± 0.4 μ; Rutin, 29.2 ± 0.4 μ; AuNPsR, 32.1 ± 0.3 μ), outer nuclear layer (CMC, 30.7 ± 0.2 μ; Rutin, 39 ± 0.7 μ; AuNPsR, 45.1 ± 0.5 μ), bacillary layer (CMC, 20.2 ± 0.1 μ; Rutin, 25.5 ± 0.1 μ; AuNPsR, 34.6 ± 0.2 μ). Insulin administration increased the width (8.2 ± 0.2 μ) of the ganglion cell layer compared to both CMC and Rutin, together with a slightly higher value (20.1 ± 0.1 μ) in the inner nuclear layer, when compared to CMC. In the remaining nuclear layers, when compared to CMC group, subjects treated with insulin showed a decrease in width: outer nuclear layer, 26.4 ± 0.8 μ; bacillary layer, 17.6 ± 0.2 μ. As per the synaptic layers, all substances administered increased the width, when compared to CMC specimens: inner plexiform layer (CMC, 30 ± 0.2 μ; insulin, 40 ± 0.5 μ; Rutin, 61.4 μ ± 0.2 μ; AuNPsR, 56.5 μ ± 0.2 μ), outer plexiform layer (CMC, 5 μ ± 0.1 μ; insulin, 5.8 μ ± 0.3 μ; Rutin, 6.8 μ ± 0.1 μ; AuNPsR, 8.9 μ ± 0.7 μ). Additionally, edema formation was observed in all treatment groups, as follows: minimal for insulin, moderate for Rutin, and advanced for AuNPsR. 

### 3.6. Eye Lens TEM Investigation

#### 3.6.1. Morphology Investigation

**Figure 14:**
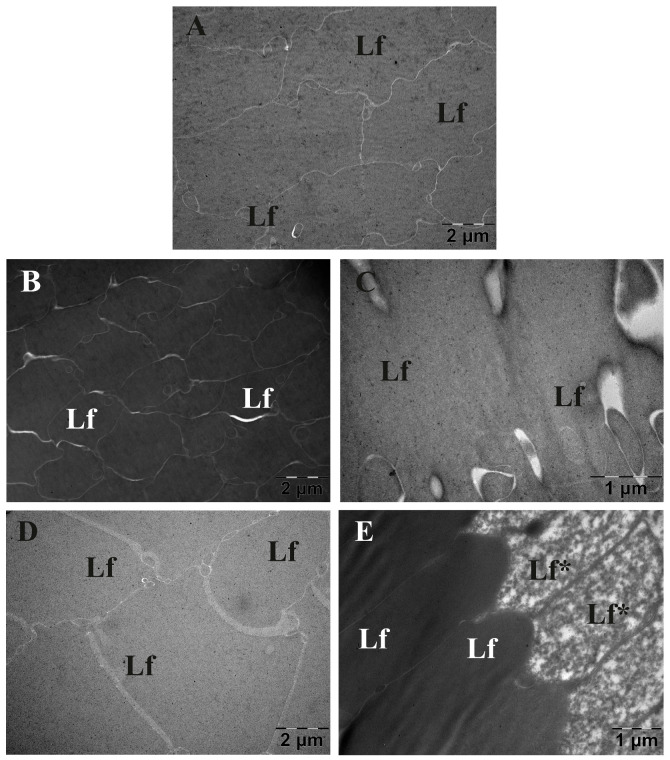
Transmission electron microscopy investigation of eye lenses from (**A**) age-matched controls, and from six-week diabetic specimens, followed by one week administration of treatments: (**B**) CMC (carboxymethylcellulose), (**C**) insulin, (**D**) Rutin, (**E**) AuNPsR (gold nanoparticles phytoreduced with Rutin); a superior electron density was observed in diabetic specimens from CMC group (**B**) and in diabetic subjects treated with insulin (**C**); diabetic animals treated with AuNPsR (**E**) showed focal lens fibre disorganisation; arrowhead points towards enlarged interfibrillar spaces (Lf, lens fibres; Lf* disorganised lens fibres).

The TEM morphological investigation of eye lenses is depicted in Figure 14. Examination of eye lenses from the Control group demonstrated a conventional ultrastructure. As such, upon inspection of randomly sampled sections, two components of higher interest can be described: primarily, tightly packed lens fibres (Lf), with a finely granular cytoplasm, and secondly, the notable delineations between them, consisting of thin, homogeneous electron lucent spaces. Regarding the areas of Lf, in CMC group, as well as in the Insulin group, most areas studied were of clear superior electron density, compared to controls. In diabetics treated with AuNPsR or Rutin, the general aspect of the tissue was comparable to that of the Control group. Additionally, a particular aspect was observed for animals with diabetes that received AuNPsR, focal Lf disorganisation. Subsequently, an enlargement of the spaces between Lf was noted in certain areas of lenses sampled from diabetic animals treated with insulin. A similar phenomenon was observed in the CMC group, and in the group treated with Rutin. Notably, in these two groups, spaces were less wide but more dispersed.

#### 3.6.2. Opacity Investigation

**Figure 15:**
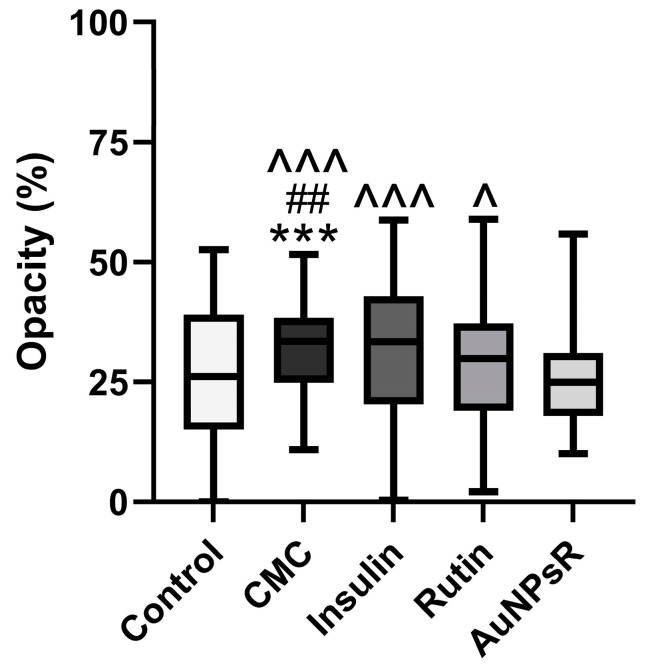
Lens opacity variation assessed on transmission electron microscopy micrographs, in controls and in rats with diabetes and treated with CMC (carboxymethylcellulose), insulin, Rutin, and AuNPsR (gold nanoparticles phytoreduced with Rutin). Parameters are expressed as minimum and maximum values, median, and interquartile range (Q1–Q3, the range between the 25th percentile and the 75th percentile), with *** *p* < 0.001 compared to Control group; ## *p* < 0.01 compared to Rutin group; ^ *p* < 0.05, ^^^ *p* < 0.001 compared to AuNPsR group.

The Kruskal–Wallis test for not-normally distributed data was employed to assess variations in lens opacity between groups. Graphed results are depicted in Figure 15. Diabetic specimens that received CMC showed a statistically significant increase in lens opacity compared to controls (*p* < 0.001). Furthermore, statistical evidence supports that in diabetic animals, the administration of insulin had similar effects to CMC, increasing opacity in the eye lens (*p* > 0.05). Rutin statistically decreased opacity compared to CMC (*p* < 0.01), while AuNPsR yielded the most favourable outcomes (*p* < 0.001). In comparison to insulin treatment of diabetics, our experiment returned a statistically stable decrease in lens opacity for rodents treated with AuNPsR (*p* < 0.001). Moreover, AuNPsR proved to have a statistical difference when compared with Rutin (*p* < 0.05). 

## 4. Discussion

Diabetes is a chronic metabolic disorder associated with endothelial dysfunction and altered vascular contractility. Its various complications, which are of clinical importance, include diabetic retinopathy and cataracts. The underlying pathophysiology is related to hyperglycaemia, oxidative stress imbalance, and inflammation of the vascular wall, with the accompanying activation of the major pathways responsible for diabetes-related damage. The present study demonstrated that DM induced the vasoconstriction of retinal arterioles, with a marked reduction in diameter. Additionally, DM increased the lipid peroxidation in eye tissues and in serum, decreased the antioxidant defence, and triggered an inflammatory response. These findings were also associated with a decrease in retinal wall thickness, and a reduction in the width of retinal layers. Additionally, DM increased lens opacity and caused marked lens fibre disorganisation. Treatment with Rutin improved the retinal arteriolar diameter, increased the antioxidant enzymatic activity and reduced the ocular levels of TNF alpha and IL-6. Consequently, Rutin administration reduced the retinal edema and preserved eye lens structure. The administration of AuNPsR improved the appearance of retinal vessels upon fundus examination, decreased MDA formation and increased the overall antioxidant capacity. However, retinal edema and a degree of disorganisation in the eye lens fibres were still observed. 

This study brings a new perspective regarding the early detection and treatment of ocular complications in DM. Additionally, this work proposed a solution to the gap in research delineated in previous articles—namely, that despite its beneficial effects in diabetic microvascular complications, Rutin has poor bioavailability and delivery, and thus lowered potency. Therefore, it was hypothesized that using gold nanoparticles as a delivery system would yield, together with an increase in systemic concentration and better ocular uptake, superior antihyperglycemic, antioxidant, and anti-inflammatory effects.

Diabetes was induced via the administration of STZ, a substance widely used due to its time and dose-dependent induction of apoptosis in pancreatic beta-cells [35]. The early development of CA and DR were closely monitored through serial examination of eye structures, using relevant studies from the literature as a guide.

Firstly, CA was considered to be in an incipient stage at the six-week mark, when, according to Muranov et al. [25], peripheral opacities were observed on anterior segment evaluation. Similarly, Aung et al. evaluated the development of CA in a rat model of post-STZ-induced diabetes and found that subcapsular CA, with characteristic spoke-like lesions, was developed only after six weeks of hyperglycaemia [36]. Moreover, they noted a gradual decline in visual acuity even before statistically significant CA formation; however, contrast sensitivity only began to decrease after nine weeks of diabetes. Thus, the studied hypothesis is further delineated, with a significant need for therapeutic strategies aimed at early-stage CA, before evident vision loss. Additionally, incipient CA development was evaluated by TEM analysis at the six-week mark, to reassure anterior segment examination findings. Similarly to our results, Majaw et al. observed a more severe disorder of lens fibres in four-week diabetic mice, compared to healthy controls, upon TEM examination [37].

The development of DR was monitored in parallel. Therefore, weekly fundus examination was used to compare diabetic subjects and healthy animals. Arteriole diameter modifications, indicative of early DR, were noted at the six-week mark. Accordingly, Lai et al. described a reduction in blood flow through the retinal arterioles between the fourth and sixth week of hyperglycaemia, in a Goto–Kakizaki rat model, a strain derived from the Wistar strain [38].

Furthermore, the therapeutic agent was chosen based on the pathophysiology of ocular diabetic complications. The mechanisms involved in incipient CA are related to lipid peroxidation, a process significantly increased in diabetes due to the imbalance between prooxidant and antioxidant systems [39]. This pathophysiological substrate justifies the use of TEM micrographs to study lens opacity. The compounds that form in the eye lens due to lipid peroxidation include dienes, a subclass of alkenes, chemical group readily reacted with OsO4, the employed TEM fixative, and contrasting agent [40,41].

Early DR has been associated with reduced retinal blood flow, as observed in an experimental model by Muir et al. [42]. This mechanism is currently explained through the hyperactivation of retinal glia by diabetes caused oxidative stress [12], which then triggers the synthesis of Ang II by the intraocular RAS, which in turn leads to a decrease in retinal blood flow by acting on AT1 microglial receptors. Additionally, the same study found that Ang II increases the production of proinflammatory cytokines through its binding to AT1 microglial receptors. This pathophysiological explanation of incipient DR is further supported by Eshaq et al. [43]. Their study demonstrated that the administration of candesartan, an AT1 receptor blocker, to diabetic Wistar rats, decreased the angiotensin converting enzyme (ACE) level, and increased the retinal blood flow. Moreover, they concluded that candesartan reduced ROS production through a decrease in p22phox levels.

In terms of treatment, insulin was utilised as a positive control because STZ destroys pancreatic beta-cells and induces type 1 diabetes (T1D). On fundoscopy examination of retinal arteriole diameter, insulin showed favourable results, restoring normal width parameters. This is an accepted action of insulin, a known vasodilator agent [44]. However, vessel diameter could be altered immediately after treatment administration, and swiftly reverted. On TEM investigation of lens opacity induced by diabetes, insulin returned results that require a more in-depth discussion, as it increased the opacity in eye lenses, similarly to vehicle administration. The discrepancy between the worsening of CA with insulin administration and its beneficial effects upon fundus examination require further detailed investigations.

The mechanism through which insulin might worsen early CA is not well known. However, Papadimitriou et al. highlighted a possible direction of research, presenting a T1D patient under an intense insulin regime, that had an elevated insulin autoantibody count at the time of bilateral CA formation [45]. Additionally, a widely accepted hypothesis is related to early worsening of DR by severe insulin therapy. Meng et al. explained this phenomenon through the overexpression of NADPH oxidase 4 enzyme activity, and thus, through the overproduction of ROS, as a result of high doses of insulin [46]. Furthermore, a possible link could exist between the early worsening of DR and early lens opacification, based on the reasoning that both CA and DR develop in the same intraocular medium, frequently *in tandem* [47]. The early worsening of DR can be explained by the osmotic force theory, which states that a forceful reduction in glucose, an osmotically active molecule, by insulin use, can lead to a shift in intraocular pressure [48]. Okamoto et al. correlated this process to an aggravated eye lens state, where a series of patients developed hyperopia because of a rapid decrease in glycaemia after insulin administration [49]. This latter explanation was consistent with the changes seen in the present study upon TEM analysis and histopathological examination. Thus, the increased interfibrillar space observed through TEM, after insulin treatment, could be caused by an alteration of intraocular pressure. This could also be correlated with the edema observed upon the retinal histopathological investigation of diabetic rats treated with insulin. Moreover, the histopathological examination yielded a smaller width of both outer nuclear and photoreceptor layers, findings which have not been previously reported, as to our knowledge. An alternative explanation for edema formation in insulin-treated rats could be related to the increased production of IL-6. It is known that IL6 mediates retinal inflammation and vascular leakage, with significant in vivo effects as early as four weeks of hyperglycaemia, as shown by Rojas et al. in a DR model of IL-6 deficient mice [50]. In addition to this, anti-IL-6 antibodies have been demonstrated to have beneficial effects in the treatment of diabetic macular edema [51].

In terms of the target therapeutic agent, an evident pattern that supports the initial claim can be observed. Regarding CA, results confirmed the present hypothesis as the best outcomes were attributed to AuNPsR treatment. On TEM investigation of lens opacity induced by diabetes, AuNPsR restored the transparency and improved the arteriolar diameter in fundoscopy. This outcome was similar to that obtained by Rutin administration alone. In fact, it can be concluded that all treatments induced vasodilation and restored the retinal arteriole diameter in DM. Moreover, Rutin and AuNPsR showed a wide range of beneficial effects, additional to those produced by insulin. Thus, upon histopathological investigation, AuNPsR restored the width of all retinal layers, lowered MDA levels and increased SOD and CAT activities.

To our knowledge, no previous study has described the benefits of AuNPsR on DR and CA. However, a similar compound, resveratrol, from the same dietary polyphenol family has been examined. Chen et al. demonstrated a statistically significant in vitro eye lens epithelial cell uptake of gold nanoparticles functionalised with resveratrol (AuNPsRes) with high biocompatibility and reduced cytotoxicity [21]. Furthermore, this study showcased the anticataractogen abilities of AuNPsRes, as it delayed the eye lens opacification upon slit-lamp examination of the studied rats. In terms of restoring transparency, the present study results are consistent with those described by Chen et al., the AuNPsR treatment showing promising benefits. This can be correlated to the favourable antioxidant effect observed in both serum and homogenised eye tissue, where AuNPsR significantly decreased MDA formation, a factor responsible for early CA onset. However, an additional focal disarray of Lf was observed upon TEM structural examination. To our knowledge, no previous study highlighted this modification after administration of gold nanoparticles. Additionally, Cosert et al. underlined the significant discrepancies between in vivo and in vitro effects of gold engineered nanomaterials, offering supplementary reasoning for the conflicting results [52]. Nonetheless, Zhang et al. reported vacuolisation of eye lenses in developing zebrafish embryos after silver nanoparticles administration, which could imply a lens deterioration by a metal-based drug delivery system [53]. Notably, this study underlined no morphological modifications in the retinas of the same specimens. Subsequently, Dong et al. highlighted the implications of AuNPsRes in treatment of DR in a Wistar albino rat model [22]. They noticed a decrease in retinal vessels permeability compared to vehicle-treated diabetic rats. This would be physiologically correlated to the effect of gold nanoparticles on arteriolar vasoconstriction. Notably, they associated these findings with low levels of vascular endothelial growth factor (VEGF), a molecule that, when targeted in early DR, yielded no significant results in restoring vision, in a recently published clinical trial [54]. Probably, the involvement of VEGF in early DR is limited, and there are multiple other factors with a vasodilator role in the retina. It is worth noting that Dong et al. evaluated retinal changes after 14 weeks of hyperglycaemia, compared to the 7 weeks in our study. Thus, VEGF-related mechanisms may be characteristic for more advanced DR. This supposition is further supported by Shi et al. and Xiao et al., who found that retinal vessel permeability was significantly increased only after the eighth week of diabetes [55,56]. Furthermore, upon histopathological examination of the retina, a characteristic of early DR was noticed, neural degeneration [57]. Retinal layers showed a decrease in width in rats with DM treated with CMC in alignment with the existing literature. Thus, Lai et al. reported lowered cellularity in both the ganglion cell layer and outer nuclear layer, coupled with a reduction in total retinal width [38]. The retinal thickness and width of all individual layers were most significantly restored in the rats treated with AuNPsR. This could be explained by the levels of ocular IL-1 beta, which were highest in the AuNPsR group. Baptista and Alveleira et al. found that IL-1 beta plays a significant role in the proliferation of retinal microglia [58]. The size of the nanoparticles studied in the present work was 15 nm, therefore, a high penetrability in the ocular tissues was obtained. Despite their benefits against neural deterioration, administration of AuNPsR induced the accumulation of retinal edema, more severe than the other treatments administered. This finding could be correlated to an IL-6-induced edema formation.

Based on the presented data, further toxicological studies are needed in order to assess the safe use of gold nanoparticles and the real benefits for the management of early ocular diabetes complications. The present study investigated the effects of a lower dose, so as to maintain feasibility to human treatment extrapolation. However, varying doses should be examined in order to obtain the ideal therapeutic cutoff.

## 5. Conclusions

Our study demonstrated that STZ administered in two doses induced DM in rats and caused the development of ocular complications after six weeks of hyperglycaemia. These pathological changes were observed both macroscopically, upon anterior segment and fundus examination, and microscopically through TEM and histopathological investigation. These changes were triggered by an oxidative stress imbalance, together with a proinflammatory status. Rutin had beneficial effects on the incipient form of cataracts and diabetic retinopathy. After one week of treatment with AuNPsR, there were noticeable antioxidant as well as anti-inflammatory effects. Morphologically, AuNPsR restored the retinal arteriole diameter, previously constricted due to hyperglycaemia. Moreover, this therapeutic agent favoured the neural restoration of the retina, alongside a reduction in eye lens opacity.

## Data Availability

Data are contained within the article.
